# Editorial: Rethinking the embodiment of language: challenges and future horizons

**DOI:** 10.3389/fpsyg.2026.1951194

**Published:** 2026-08-24

**Authors:** Valentina Cuccio, Martina Montalti, Yury Shtyrov, Vittorio Gallese

**Affiliations:** 1 Department of Ancient and Modern Civilizations, University of Messina, Messina, Italy; 2 Department of Clinical Medicine, Aarhus University, Aarhus, Denmark; 3 Department of Medicine and Surgery, University of Parma, Parma, Italy

**Keywords:** boundaries of embodiment, contextual modulation of embodied processing, embodied processing of language, embodiment (and its derivatives), replicability

## 1 Introduction

Research on the embodiment of language is entering a new phase. This renewed interest is not simply a consequence of the theoretical and methodological debates that have accompanied the field over the last decade, including concerns about replicability and the reliability of some classic findings (Morey et al., 2022). Rather, it reflects a deeper transformation in how embodiment itself is conceptualized. Instead of asking *whether* language is embodied—the fact that is no long questioned in the mainstream literature—current research increasingly seeks to determine *how, when, to what extent*, and *under which conditions* bodily resources and representations contribute to language processing, acquisition, and use. In parallel, the notion of embodiment has progressively expanded beyond mere sensorimotor simulation during word comprehension to encompass broader questions concerning the experiential grounding of meaning, the role of interaction and context, the contribution of different linguistic systems, and the dynamic relationship between bodily experience, environment, cognition, communication and learning.

The contributions collected in this Research Topic capture this ongoing shift. Together, they illustrate how the boundaries of embodied language research are being reconsidered—and moved—from multiple perspectives. Several articles extend embodiment beyond the study of isolated words and action semantics to domains that have traditionally received less attention, including grammatical structure (Garofalo et al.), sign languages (Krebs et al.), tactile communication in deaf blindness (Costain and Rose), translation (Zhou and Wang), bilingualism (Visani et al.), second language acquisition (Liu and Bau) and motor learning (Pomelova et al.). Others explore how embodied conceptual mappings operate across languages and cultures (Brglez et al.) or investigate how computational approaches, including large language models and automated metaphor analysis, can provide novel tools for studying embodied meaning in discourse (Meng et al.). Collectively, these studies demonstrate that embodiment is not merely a property of specific lexical categories but has become established as a comprehensive framework for understanding language across different modalities, linguistic systems, and communicative contexts.

The Research Topic also reflects a growing appreciation of the flexibility of embodied language processing. Rather than assuming that sensorimotor activation is an automatic consequence of linguistic input, several contributions examine the factors that modulate its emergence and functional relevance. Environment and social context (Fuchs), semantic specificity (Bolognesi) and temporal dynamics (Kogan et al.) all emerge as critical variables shaping the recruitment of bodily resources during language processing. This perspective moves beyond earlier dichotomies between embodied and amodal accounts of meaning (Arbib and Cuccio), emphasizing instead that grounding processes (in broad sense, embodiment included) are adaptive, context-sensitive, and graded.

At the same time, the Research Topic highlights important methodological challenges that continue to shape the future of the field. As illustrated by the review of articulatory suppression paradigms (De Livio et al.), embodiment research is characterized by substantial variability in experimental designs, task implementation, and reporting practices. Such heterogeneity complicates comparisons across studies and makes direct replication difficult. Addressing these issues is essential not only for improving methodological rigor but also for enabling theoretical progress, since robust evidence requires greater transparency and standardization.

Finally, the Research Topic reflects the renewed vitality of the theoretical debate surrounding embodiment. Contributions from philosophy, linguistics, psychoanalysis, and cognitive neuroscience (Arbib and Cuccio; Fuchs) argue for broader and more integrated conceptions of how bodily experiences support the emergence of meaning. These perspectives challenge traditional distinctions between concrete and abstract concepts, reconsider the relationship between embodiment and enactive approaches to cognition, and extend the discussion beyond language comprehension to include various aspects of communication, social interaction, and clinical practice. Particularly noteworthy is the growing attention to forms of language in which bodily engagement is not merely supportive but constitutive, such as sign languages (Krebs et al.) and tactile communication in congenital deafblindness (Costain and Rose), where the body becomes the primary medium through which linguistic structure and meaning emerge.

## 2 Overview of the articles

In this section we will present and briefly discuss the articles published in the *Rethinking the Embodiment of Language: Challenges and Future Horizon*s Research Topic.

Costain and Rose address language access in individuals with congenital deafblindness, proposing a tactile-signing approach grounded in the protactile movement and, drawing on Material Engagement Theory, examine two case studies of tactile communicative practice. They show that attending to tactile salience and grammaticalized reciprocity supports co-constructed signing with congenitally deafblind interlocutors, causing communication partners to shift from a transmission-based, signal-oriented model toward a dialogic, enactive one. This contribution makes a particularly poignant, almost extreme case of embodiment, demonstrating that language grounding in the body is not merely theoretical but a vital practical and clinical necessity when visual and auditory channels are absent from birth.

Arbib and Cuccio offer a theoretical contribution that directly addresses one of the central issues emerging from this Research Topic: how the role of the body in language should be conceptualized. Rather than framing embodiment as an all-or-none property of linguistic processing, they propose a graded account in which bodily and neural resources contribute to language to different degrees depending on the level of linguistic organization, communicative demands, and evolutionary history. Central to their proposal is the notion of *embrainment*, which highlights the progressive integration of bodily and brain mechanisms in the emergence and use of language, arguing that linguistic capacities cannot be reduced either to purely sensorimotor processes or to abstract symbolic representations.

De Livio et al. review a decade of studies employing articulatory suppression as verbal-interference paradigm, cataloging 147 peer-reviewed articles across five methodological dimensions (stimuli, articulation modality, rhythm, presentation format, cognitive load). They find substantial and largely unmotivated heterogeneity in how this suppression task is implemented—both as primary manipulation and as control condition—with methodological details frequently underreported, particularly when used as a control task. This contribution addresses a foundational methodological gap in embodied cognition research: articulatory suppression is, on the one hand, a key causal paradigm for testing motor-simulation accounts of inner speech, yet, on the other hand, the field's inferential strength is undermined by inconsistent, poorly standardized protocols, limiting replicability and cross-study comparability.

Garofalo et al. investigated whether object numerosity modulates the sensorimotor simulations elicited by graspable object nouns using a grasp-compatibility task. Across two experiments, they found that grammatical number alone (singular vs. plural) did not influence the grasp-compatibility effect, whereas explicit semantic quantification (e.g., *one* vs. *many*) eliminated the effect for multiple objects. These findings show that embodied language processing flexibly integrates contextual semantic information, suggesting that sensorimotor simulations are updated by salient conceptual cues rather than by grammar/morphology alone.

Markova and Enache revisit psychoanalytic theory through the lens of embodied cognition, arguing that embodied simulation, body language, and symbolization are fundamental for understanding somatic symptoms, particularly when verbal expression is limited. Drawing on findings from neuroscience and psychoanalysis, they propose that therapeutic change emerges through pre-verbal, intercorporeal interactions that gradually become symbolized. By integrating embodied simulation into psychoanalytic theory, this contribution extends embodiment beyond language comprehension to clinical practice, highlighting the foundational role of bodily interaction in the emergence of meaning.

Fuchs, in turn, develops a theoretical framework that integrates embodied cognition and enactivism to account for the experiential basis of concepts. Drawing on evidence from neuroscience, phenomenology, language acquisition, and conceptual metaphor theory, the author argues that concepts emerge from bodily interaction and participatory sense-making rather than from amodal representations. This contribution broadens the theoretical foundations of embodied language by showing that conceptual knowledge remains grounded in embodied and intersubjective experience, even in the case of abstract concepts.

Bolognesi challenges the traditional emphasis on concreteness in embodied language theories, proposing word specificity as a crucial but overlooked dimension in explaining language comprehension. Reviewing evidence from psycholinguistics and cognitive neuroscience, she argues that highly specific words may facilitate richer and more differentiated perceptual, motor, and experiential simulations than broader lexical categories, independently of their level of concreteness. This contribution refines current embodied language accounts by introducing specificity as a key factor in grounding linguistic meanings in experience.

Brglez et al. investigate methodological challenges in metaphor analysis by developing a cross-lingual approach (using English and Slovene) for identifying and disambiguating source domains in metaphorical expressions. Combining lexical resources, word embeddings, and human annotation, they evaluate the extent to which computational methods can support more transparent and systematic metaphor mapping across languages. Their findings highlight both the potential and limitations of automated approaches, showing that metaphor interpretation requires sensitivity to linguistic and conceptual context. This contribution advances the field by refining the methodological tools used to study how conceptual structures are grounded and organized through metaphor.

Liu and Bai investigate how learning Chinese pinyin influences language and cognitive development in English-speaking elementary students in a bilingual educational setting. Comparing children receiving Chinese instruction with pinyin support to peers without foreign language instruction, they examine potential cross-linguistic interference and cognitive benefits through measures of phonological awareness, vocabulary, pronunciation, and Chinese language performance. Their results show that limited pinyin exposure does not negatively affect English vocabulary or pronunciation, while its effects vary developmentally, with earlier exposure being more strongly associated with phonological abilities. This contribution expands embodied and grounded perspectives on language learning by highlighting how acquiring new linguistic systems reshapes interactions between phonological representations, cognitive development, and multilingual experience.

Krebs et al. investigate how event structure is encoded in sign language by examining the relationship between linguistic meaning and neuromotor dynamics. Using motion capture and surface electromyography (EMG), they analyze how telic and atelic verb signs differ in their movement patterns and muscle activation profiles. Their results show that event structure is reflected in systematic differences in movement deceleration, variability, motor control, and muscle co-contraction, providing physiological evidence that grammatical meaning is grounded in bodily dynamics. This contribution extends embodied language research by demonstrating that embodiment is not only a feature of spoken language comprehension but is intrinsically linked to the motor realization and grammatical organization of signed languages.

Pomelova et al. investigate the relationship between motor learning and the mirror neuron system using transcranial magnetic stimulation (TMS). Participants underwent motor training, and changes in corticospinal excitability and motor resonance were assessed to determine whether learning a novel action modulates the neural mechanisms involved in action observation and execution. Their findings provide evidence for a link between motor learning and mirror-system plasticity, demonstrating how embodied representations of actions are shaped by sensorimotor experience *per se*.

Kogan et al. provide a comprehensive review of the temporal dynamics, durability, adaptability, and vulnerability of embodied effects in language processing. Integrating evidence from behavioral, neuropsychological, neuroimaging, electrophysiological, and intracranial studies, they examine how sensorimotor systems are recruited during meaning construction and how these embodied activations evolve over time and across different conditions. They show that language-induced sensorimotor reactivations are dynamic, context-sensitive, and shaped by both experience and neural integrity, rather than being fixed consequences of word processing. This contribution advances embodied language theories by addressing key open questions about when, how long, and under which circumstances bodily grounding contributes to linguistic meaning.

Zhou and Wang address the field from an unusual perspective of embodied empathy in translation studies, focusing on how translations of traditional Chinese medicine terminology can promote readers' cognitive and emotional engagement. Drawing on embodied language comprehension theories, they argue that effective translation should not only convey conceptual meaning but also activate experiential, affective, and culturally grounded dimensions of meaning. By proposing embodied empathy as a framework for cross-cultural translation, this contribution extends embodied language research beyond comprehension and cognition, highlighting how bodily and emotional experience shape the communication of culturally embedded concepts across languages.

Meng et al. explore the use of large language models (LLMs) as a methodological tool for analyzing embodied conceptual metaphors in political discourse. Combining critical metaphor analysis with chain-of-thought prompt engineering technique, they use ChatGPT-4.0 Python environment to identify and interpret metaphorical structures in Donald Trump's speeches, examining the source domains underlying political rhetoric. Their results show that LLMs can effectively support metaphor identification and reveal how domains (such as movement, health, illness, and force) are used to construct emotional and ideological meanings, while also highlighting limitations in semantic differentiation. This contribution expands embodied language research by introducing computational approaches for investigating how embodied conceptual mappings shape discourse and social cognition.

Visani et al. investigate whether second-language (L2) action verbs engage the sensorimotor system in a manner comparable to first-language (L1) action words. In a combined behavioral and magnetoencephalography (MEG) experiment, Italian native speakers performed a semantic decisions task on action-related pictures and English L2 action verbs referring to hand and foot movements. The findings show category-specific motor system involvement during L2 verb processing, with similar neural mechanisms underlying the processing of visually and verbally presented hand actions, although L2 verbs elicited an additional processing cost compared with L1 language. This contribution supports embodied accounts of bilingual language processing by demonstrating that sensorimotor grounding extends beyond the native language and remains active during L2 comprehension.

## 3 Conclusions

Taken together, these contributions suggest that the future of embodied language research lies in defining the mechanisms, limits, and scope of embodiment. Rather than treating embodiment as a unitary phenomenon, the contributions point toward a more differentiated understanding of how bodily resources interact with linguistic, cognitive, and social processes across languages, communicative modalities, and experimental contexts. They show that embodiment is a dynamic process whose expression depends on contextual, experiential, and task-related factors.

At the same time, the Research Topic highlights that progress will require advances on multiple fronts. Theoretical accounts must become sufficiently versatile to accommodate the growing diversity of linguistic phenomena and populations under investigation, while methodological practices need greater transparency and consistency to strengthen cumulative evidence and reproducibility. The increasing dialogue between cognitive psychology, linguistics, philosophy, neuroscience, computational approaches, and clinical research further illustrates that embodied language has become an intrinsically interdisciplinary enterprise, in which multiple perspectives are essential for addressing increasingly complex questions.

Ultimately, the contributions gathered here portray a field that is redefining its own boundaries. The central challenge is to identify the multiple forms that embodiment can take, the conditions under which it emerges, and the functions it serves in language comprehension, production, and acquisition. This shift from demonstrating the phenomenon to characterizing its variability and constraints marks, in our view, the most significant development in contemporary research on embodied language.
